# Treatment of Rupture of the Uterus

**Published:** 1889-05

**Authors:** Wm. H. Wathen

**Affiliations:** Louisville, Ky., Professor of Obstetrics and Diseases of Women in the Kentucky School of Medicine; Ex-President of the Kentucky State Medical Society; Chairman of the Section on Obstetrics and Gynecology of the American Medical Association; Consulting Gynecologist to the Louisville City Hospital, etc.


### TREATMENT OF RUPTURE OF THE UTERUS.*

BY WM. H. WATHEN, M. D., OF LOUISVILLE, KY.,

Professor of Obstetrics and Diseases of Women in the Kentucky School of Medicine; Ex-President of the Kentucky State Medical Society; Chairman of the Section on Obstetrics and Gynecology of the American Medical Association; Consulting Gynecologist to the Louisville City Hospital, etc.

He said that spontaneous or accidental rupture of the uterus occurs so infrequently, during the early months of pregnancy, that he would not consider the treatment of this variety, but would confine his remarks to the treatment of rupture of the uterus during labor at or near term.

The frequency of rupture of the uterus has been variously estimated, but probably it does not occur more than once in three thousand or four thousand labors; hence a physician of large experience may never have seen a case. But this offers no excuse for neglecting the study of this important subject, and we should know all the methods of treatment that have been recommended or practiced, so as to adopt that which is followed by the best results.

The symptoms of rupture of the uterus are usually so well

*Abstract of a paper read by request before the Central Texas Medical Association, April 9th, 1889.

marked that a diagnosis can generally be made, and especially is this true where we make a careful physical examination. He had seen but one case of uterine rupture, and in this patient there was no difficulty in making a diagnosis. Rupture of the uterus cannot always be anticipated; but in pelvic deformities, or unusual obstruction to natural delivery, or pelvic or uterine tumors, rigidity of the os, bands in the vagina, hydrocephalus of the child, or in inordinate uterine contractions, cicatrices of the uterus from former wounds, and in previous disease or weakening of the uterine walls, there is danger of rupture, which may be prevented.

He could only allude to this part of the subject. An experienced obstetrician may adopt means to prevent this accident—such as dilation of the neck, removing obstructions to delivery, correcting mal-positions, the application of the forceps, and the control of inordinate uterine contractions by chloroform or morphine.

The treatment, when rupture has occurred, may be divided as follows: Expectation, extraction *per vias naturales*, and abdominal section.

The expectant treatment has resulted in but few recoveries—in 144 cases all were fatal except two; and it is probably unfortunate that any recovered, for an occasional successful result may afford the semblance of an excuse for some ignorant or timid persons not to interfere. But the science of obstetrical surgery is advancing so rapidly that he doubted if any well-civilized people will, after a while, tolerate any physician who would, by election, treat a case of rupture after the expectant method. He could imagine no instance where such treatment would be indicated, unless it be in rupture in the early months of pregnancy. Nor did he believe it wise to remove the child *per vias naturales* if all the coats of the uterus are ruptured, unless we afterward do abdominal section and remove all foreign matter from the abdominal cavity—blood, bloody serum and liquor amnii—if this is not done the woman's life is in jeopardy, for it is not possible to know the perfection of drainage into the vagina through the rent. Mundè concludes that not more than 16 per cent. of all cases treated by forceps extraction, turning and extraction, and perforation, or abandoned undelivered, recover.

This per cent. of recoveries is too high, successful cases are relatively more frequently reported than fatal ones. He did not believe that more than 10 per cent. of cases of rupture during labor, at or near term, in which abdominal section is not done, get well. If the rent is in the posterior part of the uterus, and extends into the vagina, so as to give better drainage, the woman may recover without abdominal section; but if blood, bloody serum or liquor amnii has entered into the peritoneal cavity, she would probably die. Nor is it always possible to decide how much, if any, of the discharges have gone into the cavity until we open the abdomen and examine it carefully, This examination cannot be successfully made through the vagina. He was positively in favor of laparotomy where the rent extends all through the coats of the uterus. The child may be more easily removed through the vagina, but abdominal section should then be done, and the peritoneal cavity cleansed as carefully as in best work in laparotomy for the removal of ovaries, tubes, etc.

Rupture of the uterus does not usually end fatally by immediate hemorrhage, or by primary shock, and abdominal section adds but few dangers and removes many. If perfect surgical cleanliness is observed, and the abdominal cavity made aseptic before decomposition of retained matter, or peritonitis ensues, no untoward symptom may result from the operation. The peritoneal cavity and vagina may be made aseptic by irrigation with hot water before closure of the abdominal wound.

The statistics of Trask in 1856, and of Jolly in 1870, should have determined the method of delivery. In the former, the mortality in abdominal section was 24 per cent., and when treated by other means, or abandoned, it was 71 per cent. In the latter the mortality in abdominal section was 31.6 per cent., and when treated by other methods, or abandoned undelivered, it was 86 per cent. Harris in 1880 gives the percentage of successful American puerperal abdominal sections at 53 and 11–100 per cent.

While these results positively indicate the superiority of abdominal section to any or all other methods of treatment, a further argument in its favor is that these operations were done when the medical profession knew comparatively little about the *technique* that is now so rigidly observed by all men who do good abdominal surgery.

Baudelocque gives the age of puerperal abdominal section at 121 years, and the operation was done only a few times before the year 1800. It is probable that the first successful case was published by Thibaut des Bois, of Mans, in 1768. M. Labron operated twice successfully on the same woman, in 1775 and 1776. The first operation in this country was done by Dr. Dougal and Dr. Thomas, of Pennsylvania, in 1827, and the second by Dr. Delafield, of New York, in 1828. But in no operation before 1878 was the uterus removed, or the uterine wound sutured. In 1878, Dr. Prèvot, of the Imperial Foundling Hospital, of Moscow, Russia, extirpated a ruptured uterus; the woman died of hemorrhage caused by rapid softening and involution of the tissues included in the ligature. In 1879, Dr. J. N. McCormack, of Bowling Green, Kentucky, sutured the wound in the uterus; the woman recovered and is now living.

It is hardly necessary to mention that nearly every recognized authority in obstetrics or gynecology recommends abdominal section in rupture of the uterus. His special purpose in writing this paper was to consider the best and the most successful method of treating the uterus when the abdominal cavity had been opened. If the cavity is closed without suturing the uterine wound, the blood and the lochia will probably get into the peritoneal cavity through the rent and cause death by septic peritonitis. The uterine laceration may be sutured after the fashion observed in the improved Cæsarean section, or the uterus may be amputated at the supra-vaginal neck, and the pedicle treated in the lower angle of the abdominal incision. But the torn edges are usually so jagged or irregular that it is difficult to suture the uterine muscularis, and to apply the sero-serous sutures, so as to prevent blood or lochia getting into the peritoneal cavity, or to get union of the torn edges. This may be done where the tissues of the uterus are healthy, and the tear regular and even.

Amputation of the uterus is especially indicated where there is retention of tissue elements in fatty degeneration due to malnutrition, or to retention of elements or substances that would poison the blood, such as gangrene, putrid fœtus, etc. The amputation, if correctly done, does not increase the dangers, the mischief being already done by rending of the uterine tissues and hemorrhage into the peritoneal cavity, and the profound shock of the nervous system will often be exaggerated by further hemorrhage if we delay. The amputation must be done as an operation of election, for if performed as a *dernier resort* we must not expect success. All the puerperal changes are against the late operation; for in the absence of shock, adventitious sacs, plastic adhesions, etc., will have formed, will prove troublesome and will prevent success. The operation, if done after the most approved fashion is, relatively, full of simplicity, and requires but few instruments or appliances. The abdominal incision should be made in the middle, below the umbilical line, long enough to admit the hand, and allow the child to be drawn away. When the child and the placenta are removed, throw a gum drainage tube over the fundus of the uterus and bring it down nearly to the vaginal attachment, being careful not to include the intestines. If gum drainage tube or ligature cannot be obtained, we may substitute a strong cord made of silk, cotton or flax thread. The ligature should be drawn as tightly as possible and tied firmly. The uterus may then be drawn out of the abdomen and amputated at a half to three-fourths of an inch above the ligature. Knitting needles should be introduced through the ligature and through the cervix, and the pedicle fixed in the lower angle of the abdominal incision, and touched with perchloride of iron, after the fashion of Tait. The abdomen may then be cleansed, sutured and closed after the usual fashion, and the external dressing applied. However, if the laceration extends into the vagina, and cannot all be closed by the ligature around the cervix, it would be a wise precaution to drain through the vagina by introducing a gum drainage tube through the rent into the peritoneal cavity before closing the abdominal incision. The tube should be introduced from the abdominal cavity through the rent and out through the vulva.

